# Recurrent intestinal intussusception due to ileal angiolipoma: an uncommon, rare, and potentially life-threatening entity: a case report

**DOI:** 10.3389/fonc.2025.1680934

**Published:** 2025-10-08

**Authors:** Zheng Shu, Zhe Li, Shengnan Lin, Haihua Yu

**Affiliations:** ^1^ Department of General Surgery, The First Affiliated Hospital of Shandong First Medical University & Shandong Provincial Qianfoshan Hospital, Jinan, China; ^2^ Shandong First Medical University & Shandong Academy of Medical Sciences, Jinan, China; ^3^ Department of Emergency Medicine, The First Affiliated Hospital of Shandong First Medical University & Shandong Provincial Qianfoshan Hospital, Jinan, China; ^4^ Department of Endocrinology and Metabology, The First Affiliated Hospital of Shandong First Medical University & Shandong Provincial Qianfoshan Hospital, Shandong Institute of Nephrology, Jinan, China

**Keywords:** angiolipoma, intussusception, rare pathological type, cancer, case

## Abstract

Angiolipoma, a rare benign intestinal tumor, is primarily diagnosed through abdominal imaging and pathological examination. Intestinal angiolipomas frequently cause intussusception, necessitating prompt surgical resection. This paper reports a 62-year-old female patient admitted to the First Affiliated Hospital of Shandong First Medical University with “unprovoked paroxysmal abdominal pain for 3 months.” Preoperative diagnosis indicated ascending colonic intussusception secondary to ileal angiolipoma. Pathological examination following surgical bowel resection confirmed the lesion as an (ileal) angiolipoma. Given the rarity of this pathological entity in the intestinal tract, we present this case.

## Introduction

Angiolipoma is a distinct benign adipose tissue tumor (1), composed of mature adipocytes and abnormally proliferating blood vessels (predominantly small-caliber). It most commonly occurs in subcutaneous tissues (2), particularly the forearms (near the elbow region), trunk (back, shoulders, abdomen), and lower extremities (thighs, calves). Involvement of the breast, paravertebral region, or gastrointestinal tract is exceedingly rare (3–5). Over the past decade, only six articles have reported cases of angiolipomas occurring in the gastrointestinal tract (4, 6–10). The low incidence rate poses challenges for the management of this case. This case report describes a 62-year-old female patient who presented with CT findings suggestive of intussusception involving the ascending colon and ileocecal region. Following endoscopic reduction, the intussusception recurred rapidly, necessitating partial small bowel resection with anastomosis. Serial abdominal computed tomography (CT) imaging demonstrated temporal evolution of the lesion. Histopathological examination of the resected bowel specimen confirmed the rare diagnosis of an ileal angiolipoma. This paper will offer new insights into the diagnosis and treatment of ascending colon intussusception caused by ileal hemangioma.

## Case report

**Figure 1:**
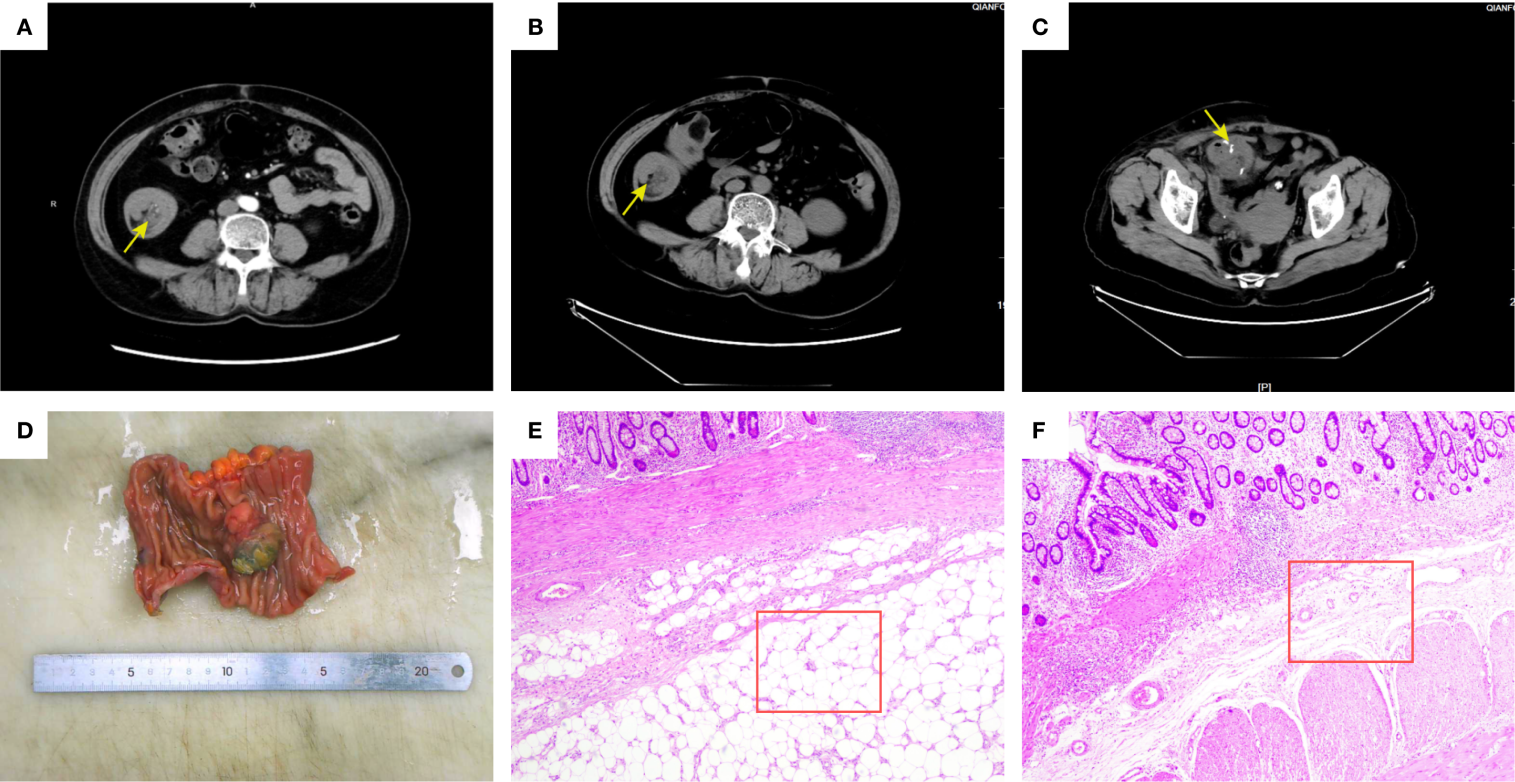
Abdominal CT imaging findings and pathological analysis of resected bowel segments. **(A)** Pre-endoscopic reduction CT: Axial view showing intussusception of the ascending colon (yellow arrow); **(B)** Post-endoscopic reduction CT: Recurrent intussusception (yellow arrow) with mesenteric fat incarceration; **(C)** Post-laparoscopic resection CT: Surgical site showing anastomotic bowel (yellow arrow) with mild wall thickening; **(D)** Macroscopic specimen shows a well-defined submucosal mass protruding into the lumen with surface ulceration; **(E)** Microscopic observation (HE stain, 40×): Submucosal angiomyolipoma composed of mature adipose tissue and proliferative vessels (red box); **(F)** Microscopic observation (HE stain, 40×): Normal submucosal intestinal tissue (red box).

A 62-year-old woman presented to The First Affiliated Hospital of Shandong First Medical University in February 2025 with a 3-month history of paroxysmal abdominal pain, exacerbated over the preceding 4 days. Her medical history included renal cyst unroofing decortication in 2018, with no family history of cancer. Physical examination revealed mild abdominal distension without visible bowel patterns or peristalsis, abdominal wall tension and resistance, tenderness with rebound tenderness; liver and spleen were non-palpable, Murphy’s sign was negative, no costovertebral tenderness was elicited, bowel sounds were slightly hyperactive (6-8/min), and shifting dullness was absent. Pre-admission gastroscopy showed no significant abnormalities. Admission lower abdominal CT demonstrated an irregular structure in the ascending colon with adipose tissue invagination into the lumen, irregular wall thickening, and no enlarged pericolonic lymph nodes (**Figure 1A**). Following clinical assessment, endoscopic reduction of the intussusception was performed. However, a repeat CT one day post-reduction revealed tortuous bowel loops in the ascending colon and ileocecal region, a double-tube sign in the ascending colon with mesenteric fat herniation, associated wall thickening, and pericolonic fat stranding, suggesting recurrent ascending colonic intussusception (**Figure 1B**). Given the confirmed diagnosis and prompt recurrence after endoscopic reduction, laparoscopic partial small bowel resection was undertaken. Intraoperatively, after reducing the intussuscepted bowel, a firm mass was identified approximately 15 cm proximal to the ileocecal valve, deemed the cause of the refractory intussusception, and resected for pathology. Postoperative CT showed findings consistent with laparoscopic small bowel resection, including linear high-density opacities and patchy soft tissue densities in the subcutaneous surgical site, with mild wall thickening around the anastomosis (**Figure 1C**). Pathological examination of the small bowel specimen revealed a submucosal angiolipoma (3.7 x 3.0 x 2.7 cm) with surface ulceration, located in the submucosa compressing the underlying muscularis propria downward, with uninvolved resection margins (**Figures 1D–F**). Immunohistochemistry was positive for CD34 (vascular component) and negative for HMB-45 and Melan-A.

## Discussion

Lipomas represent the most common benign mesenchymal tumors. They are broadly categorized into simple lipomas, composed solely of mature adipocytes, and fibrolipomas, which contain an admixture of adipose and fibrous tissue (11). Within the intestines, angiolipomas predominantly arise in the submucosa or subserosa, with a higher incidence in the small bowel compared to the colon (12). To evaluate the clinical relevance of lipomas causing intussusception, a literature search was conducted using PubMed (pubmed.ncbi.nlm.nih.gov), the free public database developed by the US National Library of Medicine (NLM). Employing the search strategy (“lipoma” OR “angiolipoma”) AND “intussusception” for publications between January 2015 and January 2025, and applying predefined inclusion/exclusion criteria supplemented by manual review of reference lists, 635 articles related to “lipoma” were identified. Of these, 22 specifically addressed “angiolipoma”, and only 6 reported cases occurring in the gastrointestinal tract (4, 6–10). Most gastrointestinal angiolipomas are small (< 2 cm) and asymptomatic. However, when tumors exceed 2 cm in size or undergo hemorrhagic rupture, they can precipitate gastrointestinal bleeding, intestinal obstruction, or intussusception (13). Intussusception occurs precisely because the angiolipomatous mass acts as a “lead point,” propelled by peristalsis into the distal bowel lumen (14).

The pathophysiological mechanism by which ileal lipomas cause ascending colon intussusception is now largely understood (15–17). This pathological process begins with the ileal lipoma acting as a local inflammatory stimulus and fixation point, initially triggering asynchronous contraction and hyperperistalsis in the proximal ileum. This abnormal peristalsis, using the tumor as a fulcrum, propels the terminal ileum into the ascending colon. During this process, the physiological anchoring effect of colonic peristalsis in the ascending colon provides essential resistance, directly facilitating the occurrence of ileo-colonic intussusception. The invagination immediately causes mechanical intestinal obstruction, leading to proximal intestinal dilation, fluid and gas accumulation, and a rapid increase in abdominal cavity volume, ultimately resulting in intra-abdominal hypertension. Intra-abdominal hypertension is not merely a consequence but actively exacerbates intestinal wall edema and compression of mesenteric vessels. This creates a vicious cycle where the compression of the intussusception sheath mutually intensifies, collectively leading to severe pathological outcomes such as intestinal ischemia and necrosis.

The etiology of intussusception in adults differs fundamentally from that in children. Over 90% of adult cases have an identifiable pathological cause, broadly categorized as neoplastic or non-neoplastic (18). Non-neoplastic causes account for only 30–40% of cases, including anatomic abnormalities (e.g., Meckel’s diverticulum, intestinal adhesions) and inflammatory or infectious processes (19–21). In contrast, neoplastic factors constitute 60–70% of etiologies, encompassing both malignant tumors (colorectal carcinoma, lymphoma, or metastatic cancer) and benign neoplasms (lipomas, gastrointestinal stromal tumors, or adenomatous polyps) (21–24). Current management primarily involves surgical intervention. Comprehensive preoperative evaluation typically identifies the underlying cause, including the precise location of intussusception and presence of a pathological “lead point (25). The initial surgical step involves manual reduction of the intussuscepted bowel segment, followed by assessment for ischemic bowel necrosis and underlying tumor pathology. For benign tumors, surgical resection is generally curative. Reported recurrence rates following resection of benign lead points are less than 5% (12). Gastrointestinal angiolipomas are exceptionally rare. Dr. Juan Sun et al. aggregated all reported cases over the past two decades, identifying only 30 documented instances. Their analysis revealed that these tumors predominantly affect middle-aged and elderly males, with the ileum and colon being the most common sites. Notably, small bowel involvement exceeds colonic occurrence (12). Abdominal pain presents non-specifically; however, angiolipomas in the upper gastrointestinal tract typically manifest with hemorrhage, whereas those in the lower tract primarily cause pain and obstruction. Remarkably, during follow-up extending to 10 years post-resection, no recurrences were documented among the 30 patients. The diagnostic challenge lies in the non-specificity of abdominal symptoms, underscoring the critical role of abdominal CT. In the present case, the patient’s longstanding gastric history initially directed evaluation toward upper GI pathology, leading to gastroscopy alone. Crucially, abdominal CT was not performed at initial symptom onset. Consequently, surgical intervention was delayed until bowel necrosis developed. While resection was ultimately performed, earlier imaging could have prevented progression to transmural compromise and mitigated risks of hemorrhagic shock or intra-abdominal infection.

In summary, ileal angiolipoma represents a rare yet treatable etiology of adult intussusception. In the present case, preoperative CT identification of a fat-density mass with vascular enhancement proved crucial for differentiating this benign lesion from malignancies such as gastrointestinal stromal tumors (GISTs) or lymphoma. Complete surgical resection remains the gold standard for preventing recurrence and complications. Clinicians should include angiolipoma in the differential diagnosis of adults presenting with occult GI pain or unexplained intussusception, particularly when imaging demonstrates fat-containing lesions. The accumulation of additional cases remains necessary to elucidate its pathogenesis and optimize management strategies.

## Data Availability

The original contributions presented in the study are included in the article/supplementary material. Further inquiries can be directed to the corresponding author.
